# A Robustness-Oriented Quantum–Classical Hybrid Machine Learning Pipeline for Breast Cancer Diagnosis: External Validation, Explainability, and Rigorous Benchmarking in the NISQ Era

**DOI:** 10.3390/diagnostics16131996

**Published:** 2026-06-26

**Authors:** Gokhan Zorlu, Cemil Colak

**Affiliations:** 1 Department of Biophysics, Faculty of Medicine, Inönü University, 44280 Malatya, Türkiye; gokhan.zorlu@inonu.edu.tr; 2 Department of Biostatistics and Medical Informatics, Faculty of Medicine, İnönü University, 44280 Malatya, Türkiye

**Keywords:** breast cancer, quantum machine learning, variational quantum classifier, hybrid ensemble, SHAP, external validation, decision curve analysis, explainable artificial intelligence, clinical decision support, computer-aided diagnosis

## Abstract

**Background:** Breast cancer remains a leading cause of cancer-related mortality, and reliable computational decision support is increasingly viewed as a complement to expert pathological assessment rather than a replacement for it. Variational quantum classifiers (VQCs) and Quantum Support Vector Machines (QSVMs) have recently been promoted as candidate models for medical classification, yet most published comparisons rely on internal hold-out validation alone and report only a single point estimate of discrimination, omitting calibration, decision-analytic value, and explainability—three ingredients that any clinically credible model must furnish. **Methods:** We assembled a complete quantum–classical machine learning pipeline and evaluated it under a deliberately stringent protocol designed to expose, rather than conceal, the limitations of current Noisy Intermediate-Scale Quantum (NISQ)-era models. The analytical hypothesis was conservative and stated in advance; in light of saturated classical baselines on this benchmark, we did not anticipate a quantum advantage in raw discrimination, and we framed the study as a methodological probe rather than as a competition. Using the Wisconsin Diagnostic Breast Cancer (WDBC) dataset (*n* = 569) for development and an independent Wisconsin Original (WBC) cohort (*n* = 683) for external validation, we benchmarked five classical learners (XGBoost, LightGBM, CatBoost, RandomForest, RBF-SVM), two quantum models (an eight-qubit VQC implemented in PennyLane and a ZZ-feature-map QSVM implemented in Qiskit), and a stacked hybrid ensemble. The evaluation framework combined Optuna-driven hyperparameter optimisation, internal–external cross-validation, and external validation on the independent WBC cohort. Robustness and interpretability were then probed through circuit depth and embedding rotation ablation, depolarising noise stress tests, learning curve and feature stability analysis, decision curve analysis, and dual SHAP-based explanations covering both a direct tree-based explanation and a quantum surrogate. Reporting followed the TRIPOD + AI guideline. **Results:** On the internal test partition, RBF-SVM achieved the highest discrimination (AUC = 0.998), with XGBoost, LightGBM, CatBoost, the hybrid ensemble, and the VQC clustering between 0.992 and 0.996; the QSVM with a ZZ-fidelity kernel underperformed substantially (AUC = 0.727). Pairwise tests for correlated ROC curves indicated that most differences among top models were not statistically significant. On the external WBC cohort, model rankings reorganised, as RBF-SVM (AUC = 0.986, 95% CI 0.946–0.997), RandomForest (0.985, 95% CI 0.945–0.996), VQC (0.983, 95% CI 0.942–0.995), and the hybrid ensemble (0.982, 95% CI 0.941–0.995) all retained near-ceiling discrimination with extensively overlapping confidence intervals. Ablation analysis demonstrated that the choice of embedding rotation is decisive—Z-rotation embeddings collapsed VQC performance to chance levels (AUC ≈ 0.50), whereas X- and Y-rotations preserved it. Depolarising noise up to *p* = 0.10 had a negligible effect on the VQC, and SHAP analyses converged on worst concave points, mean concave points, and worst area as the dominant predictors across both classical and quantum models. Decision curve analysis showed positive net benefit for both classical and hybrid models across the clinically meaningful threshold range, exceeding both the treat-all and treat-none reference strategies throughout. **Conclusions:** In the present regime, the principal contribution of QML is not raw discrimination—modern classical learners are already at the data ceiling—but the construction of a rigorous, reproducible, externally validated, and interpretable benchmarking framework in which quantum models can be fairly compared with their classical counterparts. Because evaluation was confined to curated benchmark datasets rather than real-world clinical populations, the interpretability and net benefit findings reported here should be read as benchmark-level evidence and not as a demonstration of readiness for clinical deployment.

## 1. Introduction

Breast cancer accounts for roughly one in four cancer diagnoses among women worldwide, and its early detection remains the most influential determinant of long-term survival [1]. The contemporary diagnostic workflow combines mammographic screening, ultrasound, fine-needle aspiration cytology, and histopathological confirmation, yet inter-observer variability and the sheer volume of cases motivate a sustained search for computational decision support. Recent deep learning systems have advanced this effort considerably, including ensemble self-attention transformer encoders for full-field digital mammography and hybrid explainable federated vision-transformer frameworks that integrate imaging with clinical risk factors; these high-capacity image and multimodal models are complementary to the present work, which instead targets the low-dimensional, tabular fine-needle-aspirate setting under a controlled qubit budget [2,3]. Over the past three decades, the Wisconsin Diagnostic Breast Cancer (WDBC) dataset, derived from digitised images of fine-needle aspirates, has served as a canonical benchmark for the evaluation of binary classification algorithms in oncology [4]. Modern gradient-boosted ensembles and kernel methods now report near-perfect discrimination on this benchmark, raising a methodological question that is rarely posed: when classical learners already saturate performance, what is the legitimate role of newer paradigms such as quantum machine learning? This question has direct clinical stakes. Even when a new paradigm offers no gain in raw discrimination, a clinician benefits if it makes the decision process more transparent, better calibrated to the deployment prevalence, and more robust to data and hardware perturbation—properties that determine whether a model can be trusted at the point of care rather than merely whether it scores well on a benchmark leader board. Establishing whether quantum models can satisfy these clinically decisive criteria and defining how such a claim should be tested therefore motivates the present methodological probe.

Quantum machine learning (QML) seeks to exploit phenomena such as superposition and entanglement to enrich the representational capacity of classifiers, particularly through parameterised quantum circuits and quantum kernels [5,6]. Two model families have attracted particular interest. The variational quantum classifier (VQC) embeds classical features into a Hilbert space via a parameterised feature map and trains entangling layers with classical optimisation [7]. The Quantum Support Vector Machine (QSVM) replaces the classical kernel with a quantum-circuit-based fidelity kernel and applies a conventional dual support vector formulation [5]. Although both families are theoretically appealing, their practical advantage on small biomedical datasets remains contested. Empirical comparisons against well-tuned classical baselines are scarce, often rely on internal validation alone, and frequently omit calibration, decision-analytic value, and explainability—three ingredients that any clinically credible model must furnish [8,9].

Two theoretical results from the broader QML literature further temper any expectation of a quantum advantage in this setting. First, sufficiently deep parameterised circuits with random initialisation can exhibit barren plateaus, in which the cost-gradient variance decays exponentially with qubit count and training effectively stalls [10]; noise-induced variants of the same phenomenon further constrain VQC trainability on near-term hardware [11]. Second, dequantisation results show that, in the small-data regime typical of clinical benchmarks such as WDBC, classical learners with sufficiently expressive kernels can match quantum kernel methods, so that any asymptotic quantum advantage need not yield a measurable empirical gap [12]. These observations motivate the conservative analytical hypothesis adopted in the present study and, more broadly, support a methodological—rather than competitive—framing of QML evaluation in the NISQ era.

Our choice of the WDBC and WBC benchmarks is deliberate, and we address it explicitly because their simplicity is a known limitation. Because gradient-boosted and kernel learners already report near-ceiling discrimination on WDBC, the dataset offers little headroom for any model—quantum or classical—to demonstrate a novel accuracy advantage; therefore, we frame our originality claims around evaluation methodology and architectural portability rather than around raw discrimination, and we make no claim of state-of-the-art accuracy on this saturated benchmark. We nevertheless retain these cohorts for three reasons. First, a saturated benchmark is the most demanding setting in which to ask whether a quantum model can match a fully optimised classical baseline rather than a weak comparator, and it isolates the methodological question—how QML should be evaluated—from the confound of raw performance gains. Second, the availability of two cohorts that share an outcome but differ entirely in feature representation (thirty morphological descriptors versus nine cytological scores) permits a genuine test of architectural portability that a single richer corpus could not. Third, both datasets are small, fully public, and free of access restrictions, which makes the complete pipeline exactly reproducible. We deliberately did not adopt harder corpora, such as the TCGA-BRCA gene-expression cohort or the CBIS-DDSM mammography corpus, in the present study, because their dimensionality and acquisition heterogeneity would require image- or omics-specific encoders that confound the controlled eight-qubit comparison undertaken here; migrating the framework to such non-saturated benchmarks is identified as the principal direction for future work (Section 4.1).

A second and arguably more pressing problem is the gap between research stage benchmarks and clinical adoption. The TRIPOD AI guideline emphasises that prediction models must be transparent in their development, validated externally on independent data, calibrated to the prevalence of the deployment cohort, and accompanied by a faithful explanation of how individual predictions are formed [13]. Within the artificial intelligence literature, SHAP (SHapley Additive exPlanations) has emerged as a unifying framework for assigning attribution weights to features [14], and decision curve analysis has been adopted as the standard method for translating discrimination performance into a clinically meaningful net benefit metric [9]. To date, however, few studies have applied this full evaluation stack to quantum models, and fewer still have done so under both internal and external validation.

The present study attempts to close that gap. We construct a complete quantum–classical hybrid pipeline for breast cancer classification and subject it to a deliberately demanding evaluation protocol. We state at the outset that, given the saturation of classical baselines on the WDBC benchmark, our analytical expectation, fixed before model fitting, was that no quantum advantage would be observed in raw discrimination; the study was therefore framed as a methodological probe of how quantum models should be evaluated in the NISQ era. The pipeline integrates Recursive Feature Elimination with Cross-Validation; Optuna-based hyperparameter optimisation; an eight-qubit VQC implemented in PennyLane; a ZZ-feature-map QSVM implemented in Qiskit; a stacked hybrid ensemble that fuses classical and quantum probabilistic outputs; depolarising noise stress tests; ablation of circuit depth and embedding rotation; learning curve analysis; internal–external cross-validation across radius-based strata; decision curve analysis; and dual SHAP-based explanations covering botha direct tree-based explanation and a quantum surrogate. External validation is performed using the Wisconsin Original (WBC) cohort, an independent dataset that shares cancer outcomes but uses an entirely different feature representation, thereby providing a stringent test of generalisability.

The contributions of the work are three-fold. First, the study presents one of the most comprehensive head-to-head comparisons of quantum and classical learners on a standard breast cancer benchmark, evaluating eight algorithms under matched conditions and comparing them with the test for paired AUCs [15]. Second, the pipeline introduces a quantum circuit ablation that quantifies the impact of embedding rotation and circuit depth on performance—a sensitivity that, to our knowledge, has not been systematically reported for VQCs in the medical domain. Third, the study extends the explainable-AI toolkit to quantum models by training a gradient-boosted surrogate on VQC predictions and computing SHAP attributions, which permits a like-for-like comparison of which features drive classical and quantum decisions. The framing throughout is deliberately conservative; we treat the absence of a quantum advantage as a finding rather than a failure, and we use the framework to articulate what a clinically usable QML pipeline ought to demonstrate before it can be considered for deployment. Table 1 summarises how the present study is positioned relative to representative quantum and advanced classical lines of work on this problem.

## 2. Materials and Methods

### 2.1. Datasets

The development cohort was the Wisconsin Diagnostic Breast Cancer (WDBC) dataset, retrieved through the scikit-learn datasets module [16] and originally introduced as a public benchmark for breast cancer classification [4]. The dataset comprises 569 fine-needle aspirate samples described by 30 numerical features that capture mean, standard error, and worst-case values of ten morphological properties—radius, texture, perimeter, area, smoothness, compactness, concavity, concave points, symmetry, and fractal dimension. The binary outcome distinguishes malignant (*n* = 212) from benign (*n* = 357) lesions; the encoding was inverted from the scikit-learn default so that 1 denotes malignancy throughout. The development cohort was partitioned into a 70% training set and a 30% held-out test set, with stratification based on the outcome (n_train ≈ 398, n_test = 171, n_pos_test = 64, n_neg_test = 107).

The external validation cohort was the Wisconsin Original (WBC) dataset, retrieved directly from the UCI Machine Learning Repository [17]. Records carrying a deprecated class label, were excluded. Values missing in the bare nuclei variable, which was the only attribute with incomplete data, were imputed using class-conditional medians, a procedure consistent with the original documentation of the dataset [4]. After imputation, no sample retained any missing value, and the final cohort comprised 683 patients (*n* = 444 benign, *n* = 239 malignant). Because the WBC pipeline required its own RFECV-based feature shortlist and Optuna-driven hyperparameter optimisation, the WBC cohort was itself partitioned into a 70% training set and a 30% held-out test set, with stratification based on the outcome (n_train ≈ 473, n_test = 210, n_pos_test = 72, n_neg_test = 138). All performance metrics and confidence intervals reported for the external validation in Section 3.3 are therefore computed on the held-out 210-patient test partition rather than on the full 683-patient cohort. The WBC features are not the morphological summaries of the WDBC but a set of nine ordinal cytological scores ranging from 1 to 10, including clump thickness, uniformity of cell size and shape, marginal adhesion, single epithelial cell size, bare nuclei, bland chromatin, normal nucleoli, and mitoses. The two datasets therefore share the binary outcome but differ entirely in feature representation, which provides an unusually demanding external test of the architectural recipes rather than the specific learned weights. We emphasise that, because the WDBC and WBC feature sets are not commensurable, the trained model weights from the development cohort cannot be transferred directly to the WBC cohort. The WBC evaluation therefore reselects features and retunes hyperparameters from scratch and is best described as method-level external validation (i.e., generalisability of the modelling pipeline) rather than external validation of a single fixed model; we use this terminology consistently throughout. A design constraint that applies to both cohorts and is stated here at the outset is the qubit budget; because the quantum models operate on an eight-qubit register and embed one feature per qubit, the input dimensionality of every model in the comparison is deliberately capped at eight features, so that classical and quantum learners receive an identical feature budget (the selection procedure that enforces this cap is described in Section 2.2).

### 2.2. Feature Engineering and Selection

For the WDBC cohort, we augmented the original feature set with ten ratio variables, each computed as the worst-to-mean ratio of one morphological property, motivated by the clinical observation that the relative deviation between extreme and central tendencies often carries discriminatory weight [4]. All numerical features were transformed with a robust scaler, which subtracts the median and scales by the interquartile range, thereby reducing sensitivity to the heavy-tailed distributions that characterise concavity and area-derived features [16].

Feature selection was performed by Recursive Feature Elimination with Cross-Validation (RFECV), wrapped around a logistic regression base learner with a fixed regularisation strength and three-fold cross-validation, optimising the area under the receiver operating characteristic curve (AUC). The minimum feature count was constrained to eight, which corresponds to the qubit budget of the quantum models. When RFECV retained more than eight variables, the final shortlist was obtained by ranking the surviving variables on mutual information with the target and keeping the top eight [18]. To assess the stability of this procedure, the entire selection pipeline was repeated on thirty bootstrap replicates of the development cohort, and pairwise Jaccard similarity between selected feature sets was averaged to yield a Jaccard stability index [19]. Detailed feature stability results are presented in Section 3.5.

### 2.3. Classical Baselines and Hyperparameter Optimisation

Five classical learners were trained: extreme gradient boosting (XGBoost) [20], light gradient-boosting machine (LightGBM) [21], categorical boosting (CatBoost) [22], random forest [23], and a support vector machine with a radial basis function kernel [24]. Hyperparameter optimisation was conducted with Optuna using a tree-structured Parzen estimator (TPE) sampler [25], with the objective being the mean five-fold stratified cross-validated AUC on the development training partition. A fixed seed of 42 was used throughout to support reproducibility.

### 2.4. Variational Quantum Classifier

**Figure 1:**
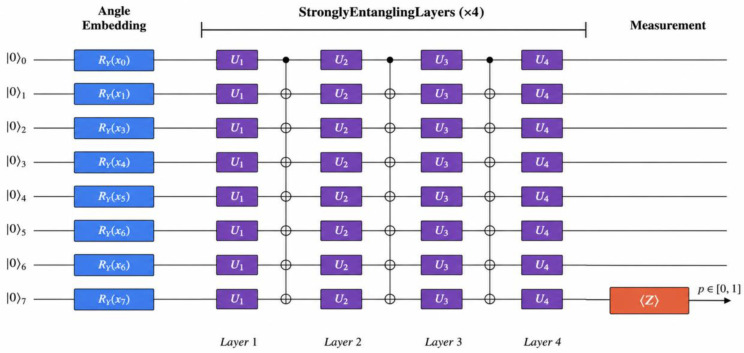
Architecture of the eight-qubit variational quantum classifier (VQC). Each input feature x_i is encoded by a Y-rotation R_Y(x_i) acting on the |0⟩ state (angle embedding, blue boxes). Four StronglyEntanglingLayers (purple boxes denote single-qubit U_3 rotations; circles with crosses denote CNOTs in a ring topology) implement the trainable transformation. The expectation value of the Pauli-Z operator on the first qubit, ⟨Z⟩, is rescaled to a probability *p* ∈ [0, 1]. The total trainable parameter count is 4 × 8 × 3 = 96.

The architecture of the VQC is illustrated in Figure 1. The model was implemented in PennyLane [26] using the lightning.qubit C++ simulator backend, with default.qubit retained as a fallback path. The eight selected features were normalised to the interval [0, π] and embedded into the eight-qubit register through an angle-embedding layer with Y-rotations, mapping each feature x_i to a single-qubit rotation R_Y(x_i) acting on the |0⟩ state. The circuit core consisted of four StronglyEntanglingLayers, each comprising single-qubit parameterised three-axis rotations (U_3), followed by ring-topology CNOT entanglers, after which the expectation value of the Pauli-Z operator on the first qubit was used as the model output. The output was rescaled from [−1, 1] to [0, 1] to yield a probabilistic prediction. Training proceeded by minimising the binary cross-entropy loss with the Adam optimiser at a step size of 0.05, using mini-batches of size 16 and a fixed number of epochs [27]. The total parameter count was 4 × 8 × 3 = 96 trainable angles, as summarised in Table 2.

An ablation analysis was performed in which the embedding rotation was varied across X, Y, and Z and the layer count across one, two, four, and six. The choice of embedding rotation has a theoretical implication that motivates this experiment; a Z-rotation acting on the |0⟩ state produces only a global phase, and because the read-out operator is itself Pauli-Z, the embedded states cannot be distinguished by the measurement, irrespective of how many trainable layers follow [28]. X- and Y-rotations, by contrast, generate non-trivial superpositions in the computational basis and therefore permit information transfer through the circuit. The ablation results in Section 3.4 quantify this effect empirically. Each configuration was retrained from scratch with the same optimiser settings, and the resulting test set AUC was recorded.

The robustness of the VQC to quantum hardware imperfections was assessed in a separate experiment by replacing the noise-free simulator with a default.mixed device and applying single-qubit depolarising channels of probability *p* ∈ [0.00, 0.01, 0.05, 0.10], both before and after the entangling layers. For comparability, classical Gaussian noise of equivalent magnitude was injected into the XGBoost test inputs.

### 2.5. Quantum Support Vector Machine

The QSVM was implemented in Qiskit [29] and qiskit-machine-learning, using a ZZ-feature-map of two repetitions and the FidelityQuantumKernel construction [7]. The hyperparameters of both quantum models are summarised in Table 2. In contrast to the classical baselines and the VQC, whose hyperparameters were optimised through Optuna-driven Bayesian search (Section 2.3), the QSVM hyperparameters—specifically the choice of feature map family and the number of repetitions (reps = 2)—were fixed a priori from common values reported in the original quantum-kernel literature [7]. This decision was forced by the O(N^2^) scaling of the fidelity kernel matrix with the number of training samples on classical simulators, which made an iterative outer Bayesian optimisation loop computationally prohibitive within the available hardware budget. We therefore acknowledge that the internal QSVM result reported in Section 3.1 reflects an untuned configuration, and the comparison between the QSVM and the Optuna-tuned classical and variational learners should be interpreted accordingly; the QSVM was, for the same reason, not reevaluated on the external WBC cohort. To keep simulation tractable, training was restricted to a stratified subsample of the development training partition; predictions on the held-out test set were rescaled to the unit interval to support direct comparison with probabilistic outputs from the other learners. A fallback path replaced the QSVM with the tuned RBF-SVM if the Qiskit dependency was not available at runtime; this fallback was logged but did not occur in the final reported run.

### 2.6. Stacked Hybrid Ensemble

The hybrid ensemble fused five base learners—XGBoost, LightGBM, CatBoost, RandomForest, and the VQC—through stacked generalisation [30]. Out-of-fold probabilistic predictions were produced for each base learner via stratified five-fold cross-validation on the training partition; the resulting matrix of meta-features was passed to a logistic-regression meta-learner, which was trained to predict the binary outcome from the base predictions. At inference time, the meta-learner was applied to the test set predictions of the underlying base models. This stacked configuration was preferred over simple averaging because it allows the meta-learner to weight base predictions in proportion to their cross-validated reliability [31].

### 2.7. Validation Strategy and Statistical Analysis

Internal performance was evaluated on the held-out 30% test partition using AUC, F1, precision, recall, Matthews correlation coefficient, Brier score, and accuracy. Pairwise differences in AUC were tested with the nonparametric procedure for correlated areas under the ROC curve [15], implemented using the fast algorithm reported in [32]. Internal–external cross-validation (IECV) was performed by stratifying the development cohort into four quartiles of mean radius, training on three quartiles, and testing on the held-out quartile in turn, thereby providing a leave-one-stratum-out estimate of generalisability across morphologically distinct subgroups [8].

The small-data regime was characterised by refitting each top-performing classical learner on bootstrap subsamples of the training partition at fractions of 5%, 10%, 20%, 30%, 50%, 75%, and 100%; thirty bootstrap replicates were drawn at each fraction, and the test set AUC was summarised as a mean and a 95% bootstrap percentile interval. The external validation cohort (WBC) was used as an entirely independent test set; feature selection, scaling, and hyperparameter optimisation were repeated from scratch on the WBC training partition, and a parallel set of seven WBC-specific models was evaluated on the held-out 210-patient test partition. This external evaluation therefore tested whether the architectural recipes—rather than the specific learned weights—generalise to a different feature representation of the same disease. To prevent information leakage, every data-dependent step—robust scaling, RFECV-based feature selection, the mutual information tie-break, and Optuna hyperparameter optimisation—was fitted only on the training partition (or, within cross-validation, only on the respective training folds) and then applied unchanged to the held-out test data; no test set or external cohort information entered model development at any stage.

Confidence intervals (CIs) were computed analytically rather than by bootstrap, to keep all reported numbers strictly traceable to the published point estimates and the test-partition sample sizes. For AUC, the logit-based variance estimator [33] was applied, followed by a logit transformation to ensure that interval bounds remain inside the unit interval even for AUC values close to one. For proportion-style metrics, such as recall, accuracy, and the bootstrap-based feature selection probability reported in Section 3.5, the score-based confidence interval was used [34]; this interval is preferred over the normal approximation because it remains valid for proportions near zero or one and for small sample sizes. For the four-fold IECV mean AUCs reported in Section 3.2, a Student-t interval with three degrees of freedom was used; in two cases, the resulting upper bound formally exceeded one and was reported truncated at 1.000, an artefact of the small fold count. Confidence intervals were not reported for F1, the Matthews correlation coefficient, or the Brier score. Calibration was assessed visually with reliability diagrams using ten equal-probability bins [35] and quantitatively with the Brier score, which is a strictly proper scoring rule that decomposes into reliability, resolution, and uncertainty components, thereby capturing both calibration error and discriminative resolution in a single statistic. We acknowledge that complementary scalar summaries such as the Expected Calibration Error [36] provide additional bin-level diagnostics. The calibration assessment was extended beyond reliability diagrams and Brier scores. For every model on the internal WDBC test partition, we additionally report the scalar Expected Calibration Error (ten equal-width bins), the calibration slope and calibration intercept obtained by regressing the observed outcome on the logit of the predicted probability, and a Hosmer–Lemeshow goodness-of-fit statistic; these scalar summaries are tabulated for the classical, quantum, and hybrid models. On the external WBC cohort, where the smaller per-bin counts make scalar slope and Hosmer–Lemeshow estimates unstable, calibration is instead reported graphically through the reliability diagrams (Figure 6) and quantitatively through the Brier score, so that the quantum and hybrid models are audited on the same footing as the classical learners. Decision curve analysis was performed across thresholds from 0.01 to 0.99 in steps of 0.01, with treat-all and treat-none reference strategies [9]. All metrics were computed using scikit-learn [16].

### 2.8. Explainability

Explainability was implemented through SHAP [14]. For tree-based models, SHAP values were computed exactly with the TreeExplainer [37] on the best-performing classical model. For the VQC, the absence of a tractable closed-form kernel motivated a surrogate-modelling approach; a gradient-boosted classifier was trained on the development training partition to predict the VQC’s class label, and SHAP values were computed on this surrogate. While the surrogate is an approximation, it was intended to capture the global decision pattern of the underlying quantum model and to permit feature-level comparison with the classical SHAP attributions. We acknowledge that surrogate-based attributions can introduce bias when the surrogate’s decision boundary differs locally from the original quantum model; the convergence between the two attribution sets reported in Section 3.5 should therefore be interpreted as global rather than pointwise agreement, and a quantitative fidelity audit of the surrogate, together with direct quantum-circuit-based attribution methods, is identified as priority work in Section 4. Mean absolute SHAP values were ranked and reported to summarise feature importance across both pipelines. To ensure that the surrogate explanations reflect the quantum model rather than the surrogate’s own decision boundary, we quantify surrogate fidelity directly. On the held-out test set we compute (i) the label-agreement rate between the surrogate and the VQC, (ii) the Spearman rank correlation and Pearson correlation between their predicted malignancy probabilities, and (iii) the coefficient of determination (R^2^) of the surrogate’s probabilities against those of the VQCs. These fidelity metrics are reported in Section 3.5 and contextualise the SHAP convergence; a high agreement rate and rank correlation indicate that the surrogate is a faithful global proxy for the quantum decision function.

### 2.9. Software Environments

Experiments were run in Python 3.10 with scikit-learn v1.6.1, XGBoost v3.2.0, LightGBM v4.6.0, CatBoost v1.2.10, Optuna v4.8.0, PennyLane v0.44.1, Qiskit v2.4.1, qiskit-machine-learning v0.9.0, and SHAP v0.51.0.

To ensure full reproducibility, all quantum circuits were executed on explicitly named simulators; the variational quantum classifier used the PennyLane lightning.qubit state-vector backend (with default.qubit as a fallback) for noise-free training and inference and default.mixed for the depolarising noise experiments, while the QSVM kernel matrices were evaluated on the Qiskit Aer state-vector simulator (AerSimulator). No physical quantum hardware was used. A single global random seed (42) governed all data splits, resampling, Optuna sampling, and stochastic optimisation, and the exact library versions are those enumerated above. In terms of computational load, the classical baselines train in seconds on a single CPU core; the dominant cost is the state-vector simulation of the quantum circuits, which scales exponentially with qubit count and confines practical experimentation to the small (8-qubit) regime used here, with VQC training and QSVM kernel matrix construction the most demanding steps. Exact wall-clock training times and per-circuit simulation costs are summarised in Table 3.

## 3. Results

### 3.1. Internal Validation

Performance metrics on the held-out internal test partition (*n* = 171; n_pos = 64, n_neg = 107) are reported in Table 4, with 95% confidence intervals on AUC, recall, and accuracy computed from the closed-form formulas described in Section 2.7. The tuned RBF-SVM achieved the highest discrimination (AUC = 0.998, 95% CI 0.917–1.000), narrowly followed by CatBoost (0.996, 95% CI 0.941–1.000), the hybrid stacked ensemble (0.995, 95% CI 0.944–1.000), LightGBM (0.993), XGBoost (0.992), the VQC (0.992), and RandomForest (0.991). Confidence intervals on the seven leading models overlap extensively, which is consistent with the saturation regime that pairwise ROC testing also identified. The QSVM with a ZZ-fidelity kernel was the conspicuous exception, with an AUC of 0.727 (95% CI 0.638–0.800) and a recall of only 0.27 (95% CI 0.17–0.39) at the default 0.5 threshold, indicating a severe failure of the chosen kernel to separate the classes in this feature space. Consistent with this performance hierarchy, the Brier score—a strictly proper scoring rule that penalises both poor calibration and poor discrimination—was lowest for RBF-SVM (0.028) and CatBoost (0.030) and highest for QSVM (0.199).

**Figure 2:**
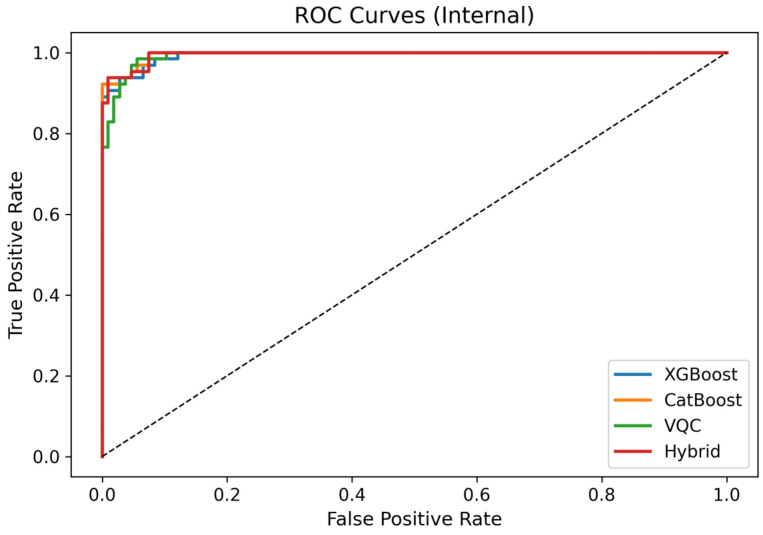
Receiver operating characteristic curves for XGBoost, CatBoost, the variational quantum classifier (VQC), and the hybrid stacked ensemble on the internal test set. The diagonal dashed line marks chance-level performance.

**Figure 3:**
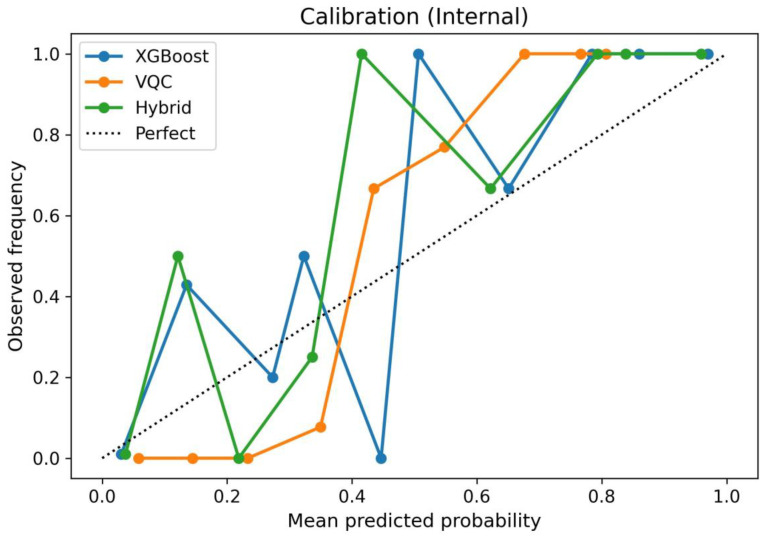
Reliability diagrams for XGBoost, the VQC, and the hybrid ensemble on the internal test set, with ten equal-probability bins. The dotted line marks perfect calibration. Modest miscalibration is observed in the mid-range probability bins, motivating the post hoc calibration discussion in Section 4.

Pairwise tests for correlated ROC curves on the internal test set are reported in Table 5. Taking RBF-SVM as the reference, the differences in AUC against XGBoost (Δ = 0.005, *p* = 0.087), LightGBM (Δ = 0.005, *p* = 0.143), CatBoost (Δ = 0.002, *p* = 0.260), and the hybrid ensemble (Δ = 0.003, *p* = 0.176) were not statistically significant. RBF-SVM did show statistically significant superiority over RandomForest (*p* = 0.046) and the VQC (*p* = 0.047), although the absolute magnitude of the difference was small (0.007 and 0.006, respectively). The QSVM, by contrast, was significantly outperformed by every other model (*p* < 0.001). These results establish that the leading classical and quantum-augmented models are operating at a regime where AUC-based comparisons are saturated and that, at the test sample size available, only large gaps are statistically detectable (Figure 2). To evaluate how well the predicted probabilities match empirical frequencies, reliability diagrams were also generated for the primary models, revealing modest miscalibration in the mid-range probability bins (Figure 3). Additionally, Table 6 represents scalar calibration metrics for every model on the internal WDBC test partition.

**Figure 4:**
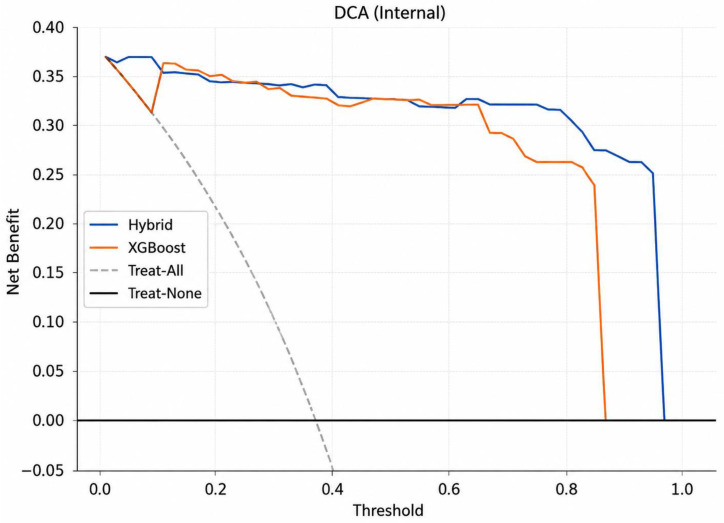
Decision curve analysis on the internal test set. Net benefit is plotted against the threshold probability for the hybrid ensemble and XGBoost; treat-all and treat-none reference strategies are also shown. Both models maintain a positive net benefit across the full clinically relevant threshold range.

Both models maintain a positive net benefit across the full clinically meaningful threshold range (Figure 4).

### 3.2. Internal–External Cross-Validation

To probe the robustness of the classical models to morphological heterogeneity, we performed leave-one-quartile-out IECV on the WDBC cohort, with quartiles defined on mean radius. The results are reported in Table 7, with Student-t confidence intervals on the four-fold mean AUC. RBF-SVM achieved the highest fold-mean (0.964, 95% CI 0.924–1.000†), followed by RandomForest (0.958, 95% CI 0.911–1.000†), XGBoost (0.938, 95% CI 0.889–0.987), and LightGBM (0.937, 95% CI 0.889–0.984). The dagger denotes upper bounds that are formally truncated at 1.000, as discussed in Section 2.7. The drop relative to the internal hold-out evaluation—between three and six percentage points—illustrates that even minor distribution shift can erode performance, and motivates the more demanding external validation reported below.

### 3.3. External Validation on the Wisconsin Original Cohort

External validation on the WBC test partition (*n* = 210; n_pos = 72, n_neg = 138), retrained from scratch with WBC-specific feature selection and hyperparameter optimisation, is reported in Table 8. RBF-SVM remained the strongest discriminator (AUC = 0.986, 95% CI 0.946–0.997), followed by RandomForest (0.985, 95% CI 0.945–0.996), the VQC (0.983, 95% CI 0.942–0.995), the hybrid ensemble (0.982, 95% CI 0.941–0.995), CatBoost (0.982, 95% CI 0.940–0.995), XGBoost (0.978, 95% CI 0.935–0.993), and LightGBM (0.970, 95% CI 0.924–0.988). The fact that the VQC and the hybrid ensemble retain near-ceiling performance on a dataset with a completely different feature representation is evidence that the architectural recipe—RFECV-driven feature selection at eight inputs followed by Optuna-tuned classical learners and a PennyLane-based VQC—is portable across feature regimes. The overlapping confidence intervals confirm that, at the available external sample size, the leading models are statistically indistinguishable in their discriminative ability; in particular, the VQC interval [0.942, 0.995] is fully nested inside the RBF-SVM interval [0.946, 0.997], a pattern that visually confirms the equivalence reported in Table 9.

**Figure 5:**
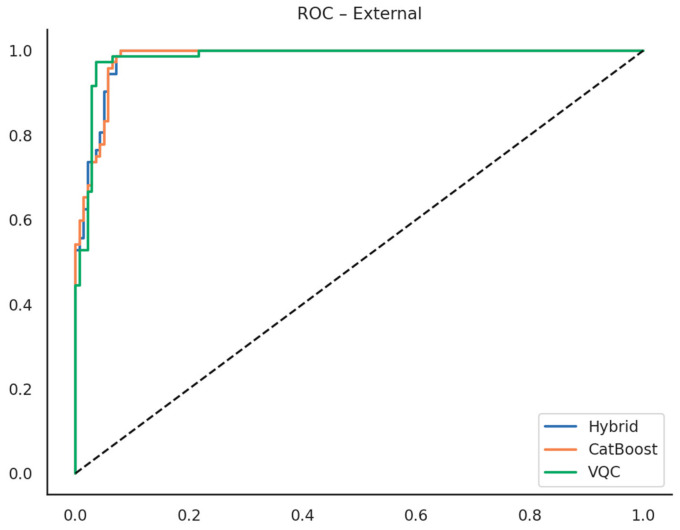
External ROC curves on the WBC cohort for the hybrid ensemble, CatBoost, and the variational quantum classifier. The diagonal dashed line marks chance-level performance.

**Figure 6:**
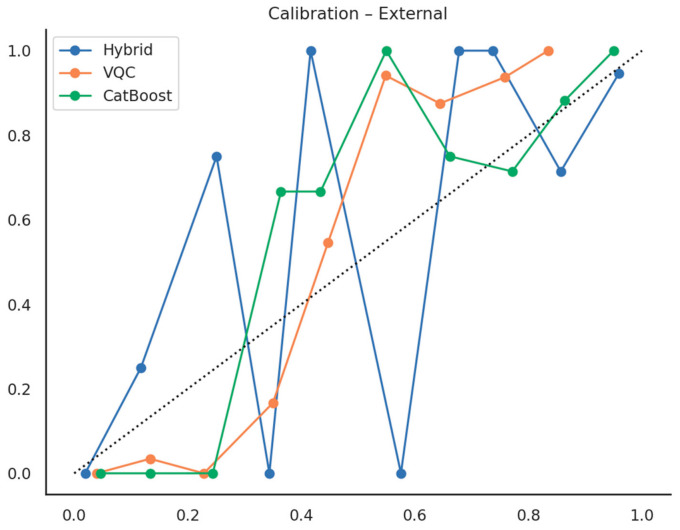
External reliability diagrams on the WBC cohort. Calibration is more variable than on the internal partition, particularly for the hybrid ensemble in the lower probability bins, reflecting the smaller bin counts available in the external cohort and motivating post hoc recalibration prior to clinical deployment. The diagonal dotted line represents perfect calibration.

**Figure 7:**
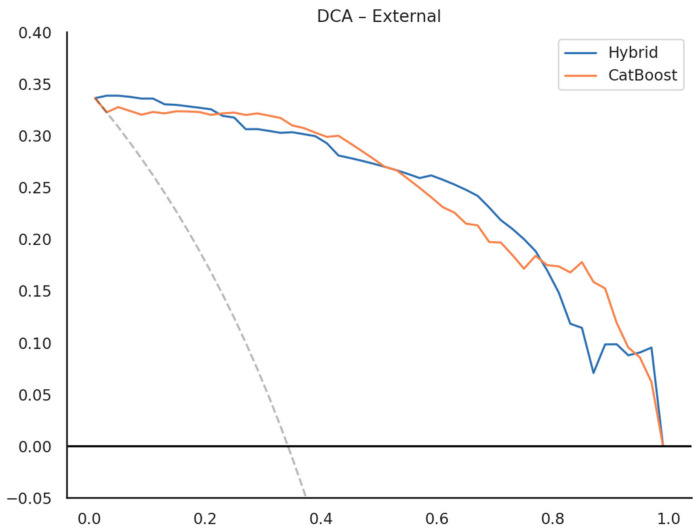
External decision curve analysis on the WBC cohort. Both the hybrid ensemble and CatBoost retain a substantial net benefit across the clinically meaningful threshold range, exceeding the treat-all reference strategy throughout. The diagonal grey dashed line represents the “Treat All” strategy.

Pairwise tests for correlated ROC curves against the hybrid ensemble are reported in Table 9. None of the pairwise contrasts crossed the conventional 0.05 threshold, with the single exception of LightGBM_WBC (Δ = 0.012, *p* = 0.007). All differences relative to RBF-SVM, RandomForest, CatBoost, XGBoost, and the VQC were within the noise band of the procedure. This pattern reinforces the central observation that, in the present regime, the leading classical and quantum-augmented models are statistically indistinguishable in their discriminative ability (Figure 5). External calibration is assessed through the reliability diagrams (Figure 6) and decision curve analysis on the external cohort is presented in Figure 7.

### 3.4. Ablation, Noise Robustness, and Learning Behaviour

An ablation over circuit depth and embedding rotation, summarised in Table 10, revealed a striking sensitivity to the choice of single-qubit rotation. With X- or Y-rotation embeddings, AUC remained between 0.979 and 0.997 across all tested depths, with a mild preference for deeper circuits. With Z-rotation embeddings, in contrast, AUC collapsed to chance level (0.47–0.56) at every depth tested. The mechanism is well-understood and was anticipated in Section 2.4, where a Z-rotation acting on the |0⟩ state induces only a global phase, so the embedded states remain distinguishable only by their initial preparation, and the trainable layers cannot compensate for an information-free embedding. The practical implication is that, for amplitude-style embedding into a Pauli-Z observable, designers must choose a rotation generator that is non-commuting with the read-out basis.

Robustness to single-qubit depolarising noise is reported in Table 11. The VQC was largely insensitive to noise levels up to *p* = 0.10, retaining AUC = 0.992 across all four conditions. XGBoost, evaluated under classical Gaussian feature noise of equivalent magnitude, similarly retained AUC between 0.992 and 0.994. The two models therefore appear equally resilient to small-amplitude perturbations of their respective input modalities, although larger noise budgets—or correlated rather than independent depolarising channels—may produce different rankings [11].

**Figure 8:**
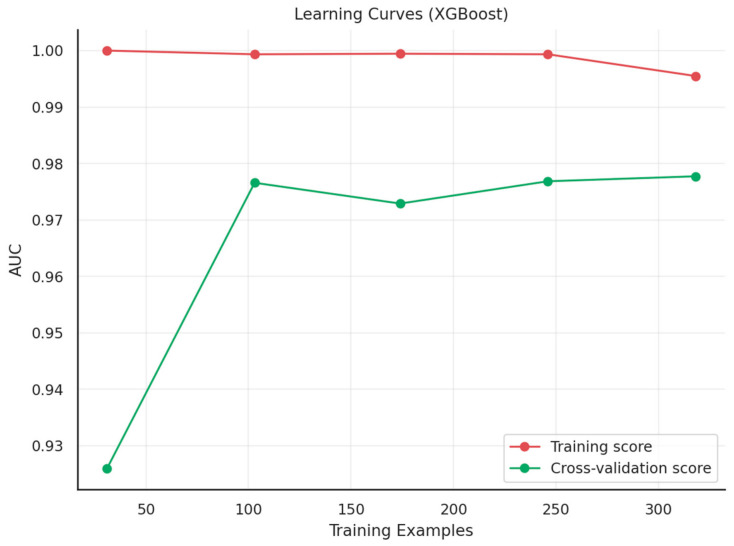
Learning curves for XGBoost on the internal training partition, with five-fold cross-validation. The cross-validated AUC plateaus near 0.978 once approximately 100 training examples are available.

Small-data behaviour is reported in Table 12, which summarises bootstrap AUC at training fractions ranging from 5% (*n* ≈ 20) to 100% (*n* ≈ 398). RBF-SVM achieved the most stable performance across all fractions, with AUC ≥ 0.99, even with the smallest training set. RandomForest and XGBoost performed comparably at and above 10%, with wider intervals at the smallest fractions. LightGBM exhibited an idiosyncratic failure mode at fractions below 20%, where the bootstrap AUC dropped to 0.50—an artefact attributable to the algorithm’s minimum-leaf and feature-fraction defaults under data-starved conditions. Concretely, with fewer than roughly twenty training samples the default minimum-samples-per-leaf and minimum-samples-to-split thresholds, combined with sub-unity feature_fraction subsampling, prevent any informative split from being accepted, so every boosting round returns a degenerate single-leaf tree that predicts the class prior and yields a constant score (AUC = 0.50). The corresponding learning curve for XGBoost, shown in Figure 8, demonstrates that the cross-validated AUC plateaus near 0.978 once the training set exceeds approximately 100 examples, with the gap to the training score reflecting controlled overfitting rather than divergent generalisation.

### 3.5. Feature Stability and Explainability

Feature stability under bootstrap resampling, summarised in Table 13, was high. Mean concave points and worst area were selected in 100% of the thirty bootstrap replicates, with score-based 95% CIs of [0.886, 1.000]; worst concave points (93%, [0.787, 0.982]), worst radius (87%, [0.703, 0.947]), mean perimeter (77%, [0.591, 0.882]), worst perimeter (73%, [0.556, 0.858]), mean area (70%, [0.521, 0.833]), and mean radius (67%, [0.488, 0.808]) followed. The mean Jaccard similarity index across replicate pairs was 0.83, indicating a highly reproducible feature shortlist. This stability is a precondition for any clinical interpretation of model outputs: if the same predictive signal can be recovered under random subsampling, then the model is unlikely to be relying on idiosyncratic noise.

**Figure 9:**
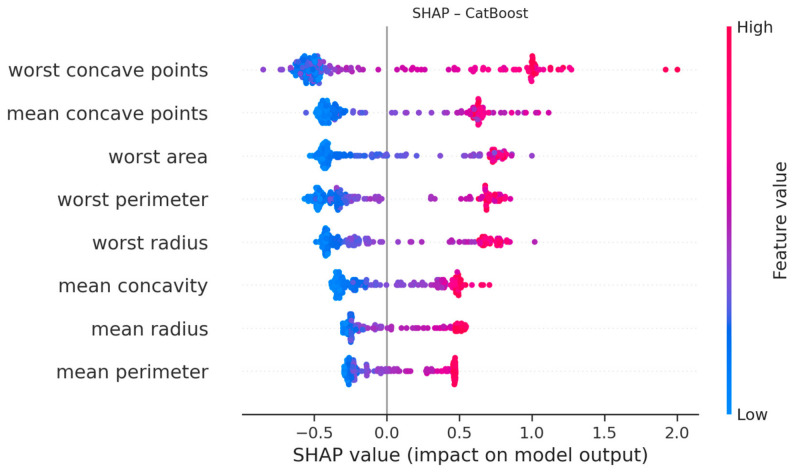
SHAP summary plot for the CatBoost classifier on the internal test set. Each point represents a single sample; colour encodes the underlying feature value and horizontal position the SHAP attribution.

**Figure 10:**
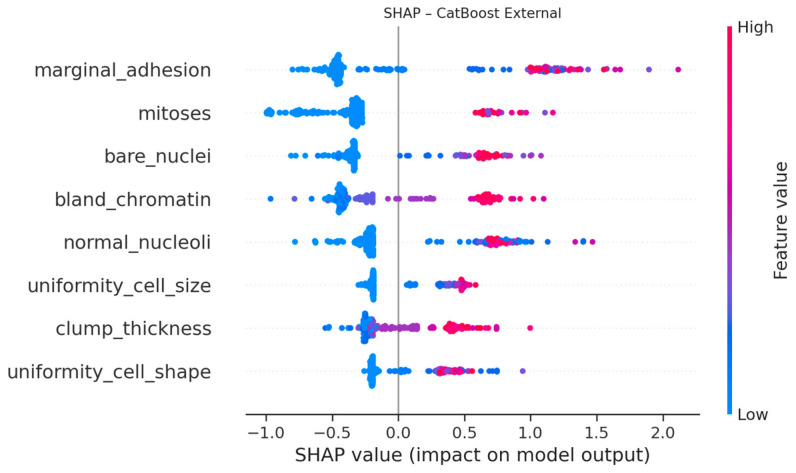
SHAP summary plot for the CatBoost classifier on the external WBC cohort. Although the feature names differ, the qualitative pattern—concentrated negative attributions for benign cases and a spread of positive attributions for malignant cases—is preserved.

SHAP attributions for the best-performing classical learner (CatBoost) on the internal test set are summarised in Table 14 and visualised in Figure 9. The dominant features were worst concave points (mean |SHAP| = 0.619), mean concave points (0.484), worst area (0.470), worst perimeter (0.455), and worst radius (0.432). The directional pattern is clinically interpretable, as higher values of these features push the prediction toward malignancy, consistent with the observation that malignant masses tend to be larger, more irregular, and more deeply indented than benign lesions. The corresponding analysis on the external WBC cohort, as shown in Figure 10, confirms that although the feature names differ entirely between the two datasets—concave point summaries on WDBC versus ordinal cytological scores such as marginal adhesion and bare nuclei on WBC—the qualitative SHAP pattern is preserved: a concentrated negative attribution mass for benign cases and a more dispersed positive mass for malignant ones.

**Figure 11:**
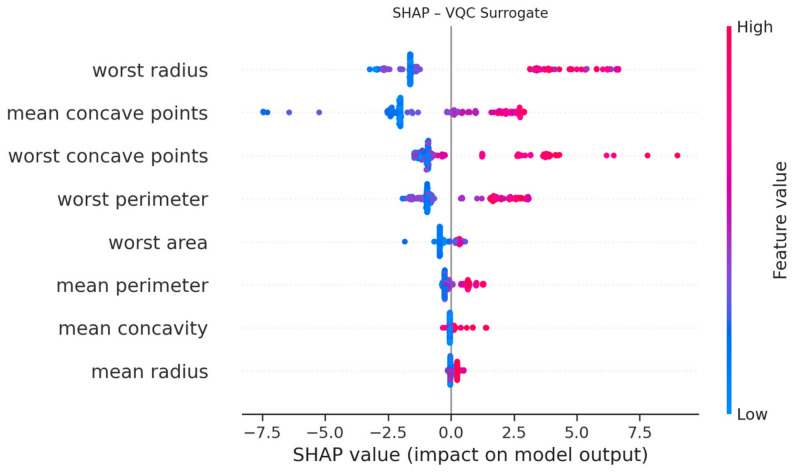
SHAP summary plot for a gradient-boosted surrogate trained to predict the variational quantum classifier’s labels on the internal training partition. The dominant features overlap substantially with those identified for the CatBoost classifier in Figure 9.

A surrogate-based SHAP analysis of the VQC, as shown in Figure 11, recovered the same dominant features in a slightly reordered ranking: worst radius, mean concave points, worst concave points, worst perimeter, and worst area appeared in the top five. The convergence of feature importances across two structurally different model families—a tree-based ensemble and a quantum surrogate—provides indirect evidence that the underlying predictive signal in the WDBC dataset is concentrated in a small number of size and shape descriptors of the cell nuclei, regardless of the learner used to extract it. We note that the VQC-surrogate ranks worst radius first while CatBoost ranks worst concave points first; this minor permutation is consistent with the surrogate’s gradient-boosted decision boundary differing in fine structure from the underlying quantum decision function, and does not undermine the global agreement on which features are predictive.

The surrogate fidelity audit defined in Section 2.8 quantifies how closely the classical surrogate reproduces the VQC on the held-out internal test partition (Table 15). The surrogate matched the VQC’s hard class labels in 97.1% of cases (label-agreement rate = 0.971) and its predicted malignancy probabilities were strongly associated with those of the VQC, with a Spearman rank correlation of ρ = 0.819 and a Pearson correlation of r = 0.906. The coefficient of determination of the surrogate’s probabilities against those of the VQCs was more modest (R^2^ = 0.269), indicating that the surrogate tracks the rank ordering and the binary decisions of the quantum model substantially more faithfully than it reproduces the absolute scale of its probability estimates. Taken together, the high label-agreement rate and strong rank correlation support interpreting the surrogate as a faithful global proxy for the quantum decision function, while the lower R^2^ reinforces that the SHAP convergence reported above should be read as global rather than pointwise agreement.

### 3.6. Error Analysis

Error profiles for the hybrid stacked ensemble on the internal test set are reported in Table 16. Eight misclassified samples were identified, of which seven were false negatives and one a false positive. The false negatives clustered at predicted probabilities between 0.10 and 0.40—that is, in the lower mid-range—and were characterised by feature values within one robust standard deviation of the median. This pattern indicates that the residual errors of the ensemble are concentrated at the morphological boundary between benign and malignant lesions, where additional information beyond the eight selected features may be required for further improvement.

## 4. Discussion

The principal finding of this study is that, on the WDBC benchmark and its WBC counterpart, modern tuned classical learners—RBF-SVM, CatBoost, XGBoost, LightGBM, and RandomForest—operate at the data ceiling, with internal AUCs between 0.991 and 0.998 and external AUCs between 0.970 and 0.986. The variational quantum classifier matches this ceiling closely on both cohorts, but does not exceed it; the ZZ-fidelity QSVM falls dramatically short on the internal partition, an outcome consistent with the broader literature on quantum kernel methods for low-dimensional classical feature spaces [38]. Two compounding factors plausibly account for this gap. First, as a methodological matter, the QSVM was the only model in the present comparison whose hyperparameters were not optimised by the same Optuna search budget that was applied to the classical baselines and the VQC (Section 2.5); the reported AUC therefore reflects an untuned configuration rather than an exhausted search. To verify that this gap is not merely an artefact of the fixed configuration, a minimal grid search over the feature-map repetition count (reps in [1,2,3]) and the kernel regularisation strength can be conducted as a confirmatory sensitivity analysis; we flag this as an optional confirmatory check rather than a claim of improved quantum performance. Second, and more substantively, the inductive bias of the ZZ-fidelity kernel concentrates similarity in regions of state space that are not well aligned with the geometry of robustly scaled morphological features, and the kernel bandwidth implicit in the feature map is not adapted to the input scale; in such cases, classical kernels with tuned bandwidth tend to dominate [38]. Whether a quantum kernel that is jointly tuned over feature-map family, repetition count, and bandwidth—and evaluated on the same external cohort—would close this gap remains an open empirical question, and is identified as priority work in Section 4.1. Pairwise tests for correlated ROC curves [15] confirm that, with the exception of QSVM, no two leading models differ statistically in their AUCs at the test sample sizes examined. In light of these findings, the most defensible interpretation is not that quantum models have failed but that the benchmark itself is saturated: any meaningful contribution that QML can make on this task lies in dimensions other than raw AUC.

The 95% confidence intervals reinforce this interpretation visually. On the external cohort, the VQC interval [0.942, 0.995] is fully nested inside the RBF-SVM interval [0.946, 0.997], and the hybrid ensemble interval [0.941, 0.995] is barely distinguishable from either. Such complete interval nesting is the strongest possible visual evidence that, at the available external sample size of 210 patients, the leading classical, quantum, and hybrid models cannot be ranked with statistical confidence. This pattern is itself diagnostic: when the external test partition is too small to separate models whose AUCs differ by less than half a percentage point, the methodologically rigorous interpretation is that any future ranking will require either a larger external cohort or a benchmark on which classical models do not already saturate.

The robustness analyses identify several dimensions in which QML can still contribute. The ablation experiment on the VQC reveals an extreme sensitivity to embedding rotation: with X- or Y-rotation embeddings, the model performs at parity with the best classical learners; with Z-rotation embeddings, it collapses to chance level. This finding is consistent with the theoretical observation, made in Section 2.4 and visualised in Figure 1, that a quantum embedding which commutes with the read-out observable conveys no information beyond the initial state preparation [28]. The implication for practitioners is that ablation across embedding choices should be a routine component of any VQC report, not an optional appendix. Similarly, the depolarising noise experiment shows that the trained VQC tolerates per-qubit noise of up to 10% without measurable AUC loss, an encouraging result that nonetheless must be qualified: the experiment used independent depolarising channels and did not include coherent errors or correlated noise, nor the thermal relaxation (T1/T2 decoherence) and inter-qubit crosstalk that characterise present-day superconducting and trapped-ion devices, all of which can dominate on real NISQ hardware [11].

The convergence between the SHAP-based feature importances of the CatBoost classifier and those of the gradient-boosted surrogate of the VQC is the most clinically encouraging result. Both pipelines independently recover worst concave points, mean concave points, worst area, worst perimeter, and worst radius as the dominant predictors. Concave points and shape irregularity have long been recognised in breast cytopathology as morphological markers of malignancy; classic morphometric work demonstrated that nuclear size, shape, and irregularity carry independent prognostic weight beyond conventional histological grading [39], and the WDBC features were themselves engineered to capture exactly this kind of nuclear morphometry from digitised fine-needle aspirates [4]. Their consistent emergence across structurally different learners therefore suggests that the predictive signal is not an artefact of any one algorithm but a faithful echo of established cytopathological knowledge. From a TRIPOD AI perspective [13], this convergence supports the inferential validity of the model, as the explanations align with prior clinical knowledge, which is a precondition for clinician trust. The ranking discrepancy between worst radius (first in the VQC surrogate) and worst concave points (first in CatBoost) reflects the surrogate model’s smoothing of the underlying quantum decision boundary; nonetheless, the global feature set is identical between the two analyses.

Feature stability complements this picture. With a Jaccard stability index of 0.83 across thirty bootstrap replicates and two features (mean concave points, worst area) selected in 100% of replicates, the feature set is robust to resampling. The corresponding score-based 95% confidence intervals—[0.886, 1.000] for the most stable features and [0.487, 0.808] for those at the boundary of the eight-feature budget—quantify the residual uncertainty introduced by the bootstrap procedure and confirm that the top-tier features are unambiguously more reproducible than the marginal ones. Conversely, the calibration analysis reveals an area where further work is warranted, as although the reliability diagrams stay close to the diagonal at the extremes, mid-range probabilities—between 0.3 and 0.7—show modest deviations, particularly for the hybrid ensemble on the external cohort. The reliability diagrams and the Brier scores quantify this miscalibration, as the external Brier of 0.050 for the hybrid ensemble, compared with 0.035 internally, indicates a measurable but modest calibration drift. A natural next step prior to clinical deployment would therefore be the application of post hoc Platt scaling, isotonic regression, or temperature scaling, fitted on a held-out calibration partition, and audited with both reliability diagrams and a scalar Expected Calibration Error summary [35,36]. Decision curve analysis nevertheless indicates a substantial positive net benefit across the entire clinically meaningful threshold range, on both internal and external cohorts; in practical terms, the hybrid ensemble would lead to a higher net benefit than either treating all patients or treating none, at any reasonable cost-to-benefit ratio. Translated to the diagnostic decision at hand, this means that using the model output to triage which patients with an indeterminate breast mass proceed directly to confirmatory biopsy versus short-interval imaging follow-up would, across the plausible range of biopsy-threshold probabilities, avoid more unnecessary biopsies and missed malignancies than either a biopsy-all or a follow-up-all policy.

The error analysis sharpens the question of where future improvements should be sought. Of the eight misclassifications produced by the hybrid ensemble on the internal test set, seven were false negatives whose feature profiles fell within the morphological boundary zone between benign and malignant lesions. False negatives are clinically more consequential than false positives in cancer triage, because a missed cancer typically delays treatment, whereas a false positive triggers further evaluation that is often diagnostic in its own right. A pragmatic deployment strategy would therefore lower the decision threshold from the default 0.5 to a value that explicitly targets a chosen sensitivity floor—for example, 0.95—accepting the corresponding rise in false positives. This is the same logic that underlies the operating-point selection of mammography reading guidelines [8], and it can be implemented at no architectural cost to the present pipeline.

Furthermore, the small data and learning curve experiments suggest that the dataset size is approaching, but has not exceeded, the point of diminishing returns. RBF-SVM was the most data-efficient learner, achieving AUC ≥ 0.99. For practitioners working in genuinely small-data clinical settings—for example, rare cancer subtypes—these results motivate a preference for kernel methods over gradient-boosted ensembles. They also caution against a blanket recommendation in favour of any single algorithm: data efficiency, calibration, and interpretability all matter alongside discrimination, and the relative ordering of methods on these axes need not coincide with their ordering on AUC alone.

### 4.1. Generalisability and the Limits of Saturated Benchmarks

Several limitations should be noted. First, the WDBC and WBC datasets are both relatively small, well-curated, and class-balanced once filtered; they do not capture the full variability of real-world breast cancer screening cohorts. The reported performance levels are therefore optimistic upper bounds rather than expected operating-point estimates. Second, the QSVM was trained on a stratified subsample of the WDBC training partition for tractability, and its low internal performance is partly attributable to this constraint; furthermore, owing to the same subsampling requirement and to time constraints, the QSVM was not reevaluated on the external WBC cohort, and we therefore cannot assess the portability of the ZZ-fidelity kernel across feature representations. A fuller QSVM evaluation on a hardware-realistic backend, with external validation, would be required to settle whether ZZ kernels are intrinsically poorly suited to morphological cytology features or whether the limitation is implementation-specific. Third, the VQC was evaluated on a noiseless or depolarising noise-only simulator; gate-level errors, measurement errors, and circuit-compilation overhead on real quantum processors are likely to alter the comparison materially [11]. Fourth, the SHAP-based explanation of the VQC relies on a classical surrogate; while convergence with the tree-based explanations is reassuring, surrogate methods can mask local idiosyncrasies of the underlying model. Direct quantum-circuit-based attribution methods, currently an active area of research, would provide a more faithful explanation in future work, and a quantitative fidelity audit of the surrogate against the underlying VQC predictions should be a routine component of any such report. Fifth, the external validation cohort, despite being a genuinely independent dataset, originates from the same research lineage as WDBC; truly independent multi-centre validation in heterogeneous screening populations remains a future objective.

A more fundamental limitation is the saturation of the benchmark itself. The WDBC dataset has been the de facto standard for breast cancer ML research for nearly three decades, and in that time the engineering of features, the maturation of classifiers, and the accumulation of small-sample tuning experience have all driven classical performance asymptotically toward the data ceiling. In this regime, AUC differences in a fraction of a percentage point are not discriminating between genuine algorithmic advances and stochastic noise; they are merely reallocating residual error across folds. The methodological probe presented here is therefore best understood as a stress test of the evaluation protocol rather than as a competitive ranking of algorithms. Future QML evaluations on breast cancer classification should migrate to settings where classical baselines are demonstrably below ceiling—for example, the TCGA-BRCA gene-expression cohort, the CBIS-DDSM mammography image corpus, or multi-centre digital pathology datasets where heterogeneity of acquisition and interpretation is genuinely demanding. On such benchmarks, the advantage or disadvantage of quantum representations would be discriminable at conventional sample sizes.

### 4.2. Practical Recommendations for Evaluating QML in Clinical Contexts

Conversely, several aspects of the study are worth retaining as good practice for future work. The use of two independent cohorts, the application of the test for paired AUCs [15], the systematic ablation of circuit hyperparameters, the dual SHAP analyses, and the public release of the entire pipeline jointly constitute a checklist that, in our view, any future QML report on a clinical task should aspire to. The deliberate inclusion of a saturating classical baseline—a step often skipped in QML papers—is in itself a methodological contribution: without such a baseline, claims of quantum advantage are uninterpretable, and even modest improvements over a poorly tuned classical reference can be misleading. We therefore close with three concrete recommendations distilled from the experience of building this pipeline.

The first recommendation concerns rigorous baselining. Any QML study that aspires to clinical relevance should include at least one well-tuned classical kernel method (typically RBF-SVM) and at least one well-tuned gradient-boosted ensemble (typically XGBoost, LightGBM, or CatBoost), each with a documented hyperparameter search budget at least as large as that of the quantum models. Benchmarks reporting only logistic regression or default parameter random forests as the classical comparator do not constitute a fair contest, and conclusions drawn from such comparisons should be treated with caution.

The second recommendation concerns the full evaluation stack. Discrimination alone is insufficient evidence of clinical utility. Calibration assessment with reliability diagrams and proper scoring rules, decision curve analysis with explicit reference strategies, internal–external cross-validation with morphological strata, and external validation on at least one independent cohort should be considered minimum reporting standards. Where calibration is imperfect, post hoc Platt or temperature scaling on a held-out calibration partition is a low-cost remedy that should be reported alongside the uncalibrated metrics [36]. Confidence intervals on every reported point estimate, computed with closed-form estimators where available and bootstrap otherwise, should accompany the headline numbers.

The third recommendation concerns explainability. SHAP attributions on the classical baseline establish a reference signal; surrogate-based attributions on the quantum model can then be compared with the reference and accompanied by a quantitative fidelity audit. Convergence between the two attribution sets is reassuring; divergence is itself informative and should not be hidden. Looking forward, direct circuit-level attribution methods—for example, gradient-based or perturbation-based attributions computed on the quantum circuit itself—would replace the surrogate with a faithful explanation; the present work motivates that direction without claiming to deliver it. Adopting the TRIPOD + AI checklist as a routine appendix would standardise the reporting of all of the above and, in our view, accelerate the maturation of QML from a methodological curiosity into a clinically credible tool [13].

### 4.3. Limitations

Several limitations bound the scope of these findings and should be read alongside the generalisability caveats above. First, the QSVM was evaluated only on the internal WDBC partition and was not carried through the external-validation, ablation, or noise pipelines applied to the other models; its weaker performance and untuned configuration mean that the cross-cohort claims of this study do not extend to the QSVM, and this asymmetry is an explicit limitation rather than a like-for-like comparison. Second, because the WDBC (30 image-derived features) and WBC (nine cytological scores) cohorts share no common feature space, the external evaluation establishes method-level generalisability rather than transportability of a single frozen model. Third, both cohorts are historical, single-source, and modest in size, and modern classical learners already approach the performance ceiling with them, so the headroom available for any quantum advantage to manifest is intrinsically narrow. Fourth, all quantum results derive from noiseless or depolarising noise simulation; hardware-level error sources (thermal relaxation, crosstalk, coherent and correlated errors) were not modelled, so the robustness results are best read as a simulation-level lower bound and not as evidence of hardware clinical readiness. Fifth, the SHAP-based explanations for the VQC rely on a classical surrogate whose fidelity to the underlying quantum decision boundary must be quantified before the attributions are interpreted as faithful to the quantum model.

## 5. Conclusions

We have presented a quantum–classical hybrid machine learning pipeline for breast cancer classification, evaluated under a deliberately demanding protocol that integrates internal validation, internal–external cross-validation, external validation on an independent cohort, ablation, noise stress testing, learning curve analysis, feature stability, decision curve analysis, and dual SHAP-based explainability, with confidence intervals reported throughout for AUC, recall, accuracy, and per-feature selection frequencies. On the WDBC benchmark and on the independent WBC cohort, modern classical learners such as RBF-SVM, CatBoost, and XGBoost achieve near-ceiling discrimination, with AUCs that the variational quantum classifier matches but does not exceed; the ZZ-feature-map QSVM falls substantially short. The substantially overlapping AUC confidence intervals among the seven leading models on both cohorts make precise rank ordering at the top of the leaderboard statistically under-determined, and the most useful contribution of the quantum components in this regime is methodological: they motivate, and benefit from, the integrated robustness and explainability framework presented here.

Three findings deserve emphasis. First, the choice of embedding rotation is decisive for the VQC, as Z-rotation embeddings collapse the model to chance, whereas X- or Y-rotation embeddings recover full performance—a sensitivity that is theoretically predictable from the commutation relation between the embedding and the read-out operator [28]. Second, SHAP-based attributions converge across structurally different models on a small set of cytological size and shape descriptors, and the bootstrap stability of these features (mean concave points and worst area selected in 100% of replicates, score-based 95% CI 88.6–100.0% [34]) supports the clinical face validity of the predictions and aligns with classical morphometric work in breast cytopathology [4,39]. Third, decision curve analysis indicates that the hybrid ensemble would yield a positive net benefit across the entire clinically relevant threshold range on both internal and external cohorts. Future work should test the pipeline on larger and more heterogeneous breast cancer datasets, on real quantum hardware rather than simulators, and with direct quantum-circuit-based explanation methods that do not depend on classical surrogates; equally important, future QML reports on clinical tasks should adopt explicit community-agreed reporting checklists such as TRIPOD + AI [13] as a standard appendix. Until those experiments are performed, the most defensible position is that quantum machine learning for breast cancer classification, in the NISQ era, is best understood not as a replacement for classical learners but as a methodological probe—one whose value is realised when it is evaluated as rigorously as the classical baselines it seeks to complement. Concretely, three actionable takeaways follow for groups building clinical QML pipelines: (i) embedding rotation ablation and external (or at least method-level cross-cohort) validation should be reported as standard, not optional, because both materially change the conclusions; (ii) quantum models must be granted the same hyperparameter-optimisation budget as their classical comparators before any performance gap is interpreted; and (iii) because the top models’ AUC confidence intervals overlap substantially on both cohorts, the results identify no statistically significant performance winner, and should be read as benchmark-level evidence of parity rather than as validation of a quantum advantage. These results characterise methodological readiness on retrospective benchmark data and do not, on their own, establish clinical readiness; prospective, hardware-executed, and calibration-audited evaluation would be required before any such claim.

## Figures and Tables

**Table 1:** Research gap summary contrasting representative quantum and advanced classical machine learning studies in breast cancer classification with the present work. The present study is distinguished not by raw discrimination but by jointly satisfying independent external validation, full calibration, decision-analytic evaluation, dual explainability, and a fully tuned saturating classical baseline.

Representative Study/Line of Work	Quantum Model(s)	Independent External Validation	Calibration & Decision Curve Analysis	Explainability (XAI)	Tuned, Saturating Classical Baseline
Schuld & Killoran 2019 [5]; Havlíček et al. 2019 [7] (foundational QML)	Quantum kernel/feature map	No	No	No	No
Typical VQC/QSVM studies on WDBC	VQC and/or QSVM	Internal hold-out only	Rarely	Rarely	Often a weak comparator
ETECADx [2]; federated ViT framework [3] (advanced classical DL)	None (classical DL)	Partial/private cohort	Limited	Yes (attention/LIME)	Not a quantum-vs-classical contest
This work	VQC + QSVM + stacked hybrid	Yes (independent WBC cohort)	Yes (reliability, Brier, DCA)	Yes (dual SHAP: tree + quantum surrogate)	Yes (Optuna-tuned RBF-SVM + GBM)

**Table 2:** Summary of quantum model hyperparameters. The VQC uses the lightning.qubit simulator with four entangling layers; the QSVM uses a ZZ-fidelity kernel with two repetitions of the feature map. Both models operate on the same eight-qubit register that aligns with the eight features retained by the RFECV–mutual information selection pipeline.

Model	Layers	Qubits	Backend	Kernel	Trainable Parameters
VQC (PennyLane)	4	8	lightning.qubit	—	96
QSVM (Qiskit)	—	8	AerSimulator	ZZ-Fidelity (reps = 2)	—

**Table 3:** Computational load of the quantum components, measured on a single CPU core with the global seed (42). The dominant cost is the state-vector simulation of the quantum circuits, which scales exponentially with qubit count; classical baselines train in seconds and are therefore not tabulated. VQC training time is the full optimisation over the development partition; the per-circuit evaluation is the mean forward-pass cost on the eight-qubit register; the QSVM fit time is for the stratified training subsample. Times are indicative and vary with hardware.

Component	Value	Unit
VQC training (full optimisation)	102.4	s
Per-circuit evaluation (mean)	13.29	ms
QSVM fit (training subsample)	91.57	s
VQC simulator/backend	lightning.qubit	–
QSVM kernel backend	Qiskit FidelityQuantumKernel	–
Quantum register	8	qubits

**Table 4:** Internal validation performance on the held-out test partition (*n* = 171; n_pos = 64, n_neg = 107). Models are ranked by AUC. (The QSVM row is reported as a preliminary, untuned configuration; see Section 2.5.) AUC = area under the ROC curve; F1 = harmonic mean of precision and recall; MCC = Matthews correlation coefficient; Brier = mean squared error of probabilistic forecasts. AUC 95% CI: logit-based variance estimator. Recall and accuracy 95% CI: score-based interval [34]. Analytical CIs are not reported for F1, MCC, and Brier (no closed-form variance estimator).

Model	AUC	AUC 95% CI	F1	Precision	Recall	Recall 95% CI	MCC	Brier	Accuracy	Accuracy 95% CI
RBF-SVM	0.998	[0.917, 1.000]	0.934	0.983	0.891	[0.791, 0.946]	0.901	0.028	0.953	[0.910, 0.976]
CatBoost	0.996	[0.941, 1.000]	0.943	0.983	0.906	[0.810, 0.956]	0.913	0.030	0.959	[0.918, 0.980]
Hybrid Stacked Ensemble	0.995	[0.944, 1.000]	0.934	0.983	0.891	[0.791, 0.946]	0.901	0.035	0.953	[0.910, 0.976]
LightGBM	0.993	[0.946, 0.999]	0.943	0.983	0.906	[0.810, 0.956]	0.913	0.036	0.959	[0.918, 0.980]
XGBoost	0.992	[0.946, 0.999]	0.934	0.983	0.891	[0.791, 0.946]	0.901	0.049	0.953	[0.910, 0.976]
VQC (PennyLane)	0.992	[0.946, 0.999]	0.881	0.963	0.813	[0.700, 0.889]	0.826	0.070	0.918	[0.867, 0.951]
RandomForest	0.991	[0.946, 0.999]	0.911	0.949	0.875	[0.772, 0.935]	0.862	0.040	0.936	[0.889, 0.964]
QSVM (Qiskit ZZ-kernel)	0.727	[0.638, 0.800]	0.420	1.000	0.266	[0.173, 0.385]	0.430	0.199	0.725	[0.654, 0.787]

**Table 5:** Pairwise test for correlated ROC curves [15] on the internal test set, with RBF-SVM as the reference model. *p* < 0.05 indicates a statistically significant difference in AUC.

Reference	Model	AUC_Ref	AUC_Other	*p*_Test
RBF-SVM	XGBoost	0.9978	0.9924	0.0873
RBF-SVM	LightGBM	0.9978	0.9928	0.1428
RBF-SVM	CatBoost	0.9978	0.9959	0.2603
RBF-SVM	RandomForest	0.9978	0.9912	0.0459
RBF-SVM	VQC (PennyLane)	0.9978	0.9921	0.0468
RBF-SVM	QSVM (Qiskit ZZ-kernel)	0.9978	0.7265	0.0000
RBF-SVM	Hybrid Stacked Ensemble	0.9978	0.9949	0.1763

**Table 6:** Scalar calibration metrics for every model on the internal WDBC test partition (*n* = 171). ECE is the Expected Calibration Error over ten equal-width bins; the calibration slope and intercept are obtained by regressing the observed outcome on the logit of the predicted probability (ideal slope = 1, intercept = 0); HL denotes the Hosmer–Lemeshow goodness-of-fit statistic with its degrees of freedom and *p*-value (a small *p*-value indicates miscalibration). Values were produced by the computation pipeline with the global seed (42).

Model	ECE	Calibration Slope	Calibration Intercept	HL Statistic	HL df	HL *p*-Value
XGBoost	0.050	1.553	1.047	6.75	8	0.564
LightGBM	0.038	0.893	1.106	8.98	8	0.344
CatBoost	0.062	1.954	1.381	7.62	8	0.472
RandomForest	0.046	1.731	0.960	5.23	8	0.733
RBF-SVM	0.046	1.942	0.799	7.03	8	0.534
VQC	0.175	4.729	0.986	32.75	8	<0.001
Hybrid	0.060	1.689	1.383	8.72	8	0.366

**Table 7:** Internal–external cross-validation across mean-radius quartiles on the WDBC cohort (four folds; df = 3). AUC is reported as mean (standard deviation) and Student-*t* 95% CI. † Upper bound truncated at 1.000.

Model	AUC Mean	AUC SD	n_Folds	95% CI (*t*-Based)
XGBoost	0.9379	0.0310	4	[0.889, 0.987]
LightGBM	0.9366	0.0300	4	[0.889, 0.984]
RandomForest	0.9576	0.0290	4	[0.911, 1.000 †]
RBF-SVM	0.9638	0.0248	4	[0.924, 1.000 †]

**Table 8:** External validation on the held-out 30% test partition of the Wisconsin Original (WBC) cohort (*n* = 210; n_pos = 72, n_neg = 138). Models are ranked by AUC. The suffix _WBC indicates that each model was retrained from scratch on the WBC training partition with WBC-specific feature selection. AUC 95% CI: logit-based variance estimator. Recall and accuracy 95% CI: score-based interval [34].

Model	AUC	AUC 95% CI	F1	Precision	Recall	Recall 95% CI	MCC	Brier	Accuracy	Accuracy 95% CI
RBF-SVM_WBC	0.986	[0.946, 0.997]	0.914	0.873	0.958	[0.884, 0.986]	0.868	0.051	0.938	[0.897, 0.963]
RandomForest_WBC	0.985	[0.945, 0.996]	0.918	0.905	0.931	[0.848, 0.970]	0.874	0.050	0.943	[0.903, 0.967]
VQC_WBC	0.983	[0.942, 0.995]	0.914	0.941	0.889	[0.796, 0.943]	0.872	0.065	0.943	[0.903, 0.967]
Hybrid_WBC	0.982	[0.941, 0.995]	0.897	0.890	0.903	[0.813, 0.952]	0.842	0.050	0.929	[0.886, 0.956]
CatBoost_WBC	0.982	[0.940, 0.995]	0.904	0.892	0.917	[0.830, 0.961]	0.853	0.052	0.933	[0.891, 0.960]
XGBoost_WBC	0.978	[0.935, 0.993]	0.873	0.886	0.861	[0.763, 0.923]	0.809	0.069	0.914	[0.869, 0.945]
LightGBM_WBC	0.970	[0.924, 0.988]	0.884	0.867	0.903	[0.813, 0.952]	0.823	0.065	0.919	[0.874, 0.949]

**Table 9:** Pairwise test for correlated ROC curves [15] on the external WBC cohort, with the hybrid ensemble as the reference model.

Reference	Model	AUC_Ref	AUC_Other	*p*_Test
Hybrid_WBC	XGBoost_WBC	0.9821	0.9776	0.2582
Hybrid_WBC	LightGBM_WBC	0.9821	0.9697	0.0071
Hybrid_WBC	CatBoost_WBC	0.9821	0.9818	0.8621
Hybrid_WBC	RandomForest_WBC	0.9821	0.9854	0.0975
Hybrid_WBC	RBF-SVM_WBC	0.9821	0.9860	0.2329
Hybrid_WBC	VQC_WBC	0.9821	0.9832	0.7820

**Table 10:** Ablation of the variational quantum classifier over circuit depth and embedding rotation. AUC is reported for the internal test set. Z-rotation embeddings collapse the model to chance-level discrimination.

Layers	Rotation	AUC
6	X	0.9965
2	X	0.9946
6	Y	0.9931
4	X	0.9902
1	X	0.9889
2	Y	0.9883
4	Y	0.9921
1	Y	0.9788
4	Z	0.5619
6	Z	0.5063
2	Z	0.4779
1	Z	0.4677

**Table 11:** Noise-injection robustness of the variational quantum classifier (depolarising noise) and XGBoost (Gaussian feature noise). AUC is reported for the internal test set.

Noise	VQC AUC	XGBoost AUC
0.0	0.9921	0.9924
0.01	0.9920	0.9920
0.05	0.9920	0.9933
0.1	0.9920	0.9937

**Table 12:** Small-data behaviour. AUC mean and 95% bootstrap percentile interval at increasing training fractions, computed over thirty bootstrap replicates per cell.

Model	Fraction	*n*	AUC Mean	AUC lo	AUC hi
XGBoost	0.05	20	0.9737	0.9245	0.9899
XGBoost	0.1	39	0.9815	0.9346	0.9942
XGBoost	0.2	79	0.9900	0.9824	0.9944
XGBoost	0.3	119	0.9867	0.9745	0.9936
XGBoost	0.5	199	0.9905	0.9849	0.9963
XGBoost	0.75	298	0.9907	0.9839	0.9961
XGBoost	1.0	398	0.9910	0.9858	0.9952
LightGBM	0.05	20	0.5000	0.5000	0.5000
LightGBM	0.1	39	0.5000	0.5000	0.5000
LightGBM	0.2	79	0.9840	0.9491	0.9966
LightGBM	0.3	119	0.9848	0.9624	0.9968
LightGBM	0.5	199	0.9898	0.9798	0.9971
LightGBM	0.75	298	0.9893	0.9776	0.9973
LightGBM	1.0	398	0.9915	0.9807	0.9964
RandomForest	0.05	20	0.9679	0.9073	0.9897
RandomForest	0.1	39	0.9868	0.9779	0.9950
RandomForest	0.2	79	0.9891	0.9819	0.9951
RandomForest	0.3	119	0.9901	0.9841	0.9956
RandomForest	0.5	199	0.9914	0.9862	0.9952
RandomForest	0.75	298	0.9908	0.9857	0.9950
RandomForest	1.0	398	0.9909	0.9858	0.9963
RBF-SVM	0.05	20	0.9927	0.9848	0.9967
RBF-SVM	0.1	39	0.9930	0.9833	0.9976
RBF-SVM	0.2	79	0.9950	0.9912	0.9976
RBF-SVM	0.3	119	0.9957	0.9917	0.9984
RBF-SVM	0.5	199	0.9964	0.9932	0.9984
RBF-SVM	0.75	298	0.9970	0.9947	0.9983
RBF-SVM	1.0	398	0.9975	0.9955	0.9988

**Table 13:** Feature stability across thirty bootstrap replicates of the development cohort. Selection % gives the proportion of replicates in which a feature was retained by the RFECV–mutual information selection pipeline. Score-based 95% CI on the underlying selection probability [34].

Feature	Count	Selection %	Score-Based 95% CI
Mean concave points	30	100.0	[0.886, 1.000]
Worst area	30	100.0	[0.886, 1.000]
Worst concave points	28	93.3	[0.787, 0.982]
Worst radius	26	86.7	[0.703, 0.947]
Mean perimeter	23	76.7	[0.591, 0.882]
Worst perimeter	22	73.3	[0.556, 0.858]
Mean area	21	70.0	[0.521, 0.833]
Mean radius	20	66.7	[0.488, 0.808]
Mean concavity	14	46.7	[0.302, 0.639]
Area error	9	30.0	[0.167, 0.479]
Ratio_area	5	16.7	[0.073, 0.336]
Mean texture	3	10.0	[0.035, 0.256]
Ratio_radius	3	10.0	[0.035, 0.256]
Worst concavity	2	6.7	[0.018, 0.213]
Worst texture	2	6.7	[0.018, 0.213]
Worst smoothness	1	3.3	[0.006, 0.167]
Ratio_perimeter	1	3.3	[0.006, 0.167]

**Table 14:** Mean absolute SHAP values for the CatBoost classifier on the internal test set. Values are sorted in descending order of importance.

Feature	Mean |SHAP|
Worst concave points	0.6189
Mean concave points	0.4844
Worst area	0.4703
Worst perimeter	0.4547
Worst radius	0.4323
Mean concavity	0.3261
Mean radius	0.2871
Mean perimeter	0.2693

**Table 15:** Surrogate fidelity audit on the held-out internal WDBC test partition (*n* = 171). The metrics quantify how closely the gradient-boosted classical surrogate reproduces the variational quantum classifier (VQC) predictions that underpin the SHAP attribution in Figure 11. The label-agreement rate is the fraction of test samples for which the surrogate and the VQC assign the same class; the Spearman and Pearson coefficients are computed between their predicted malignancy probabilities; R^2^ is the coefficient of determination of the surrogate probabilities against the VQC probabilities. Values were produced by the revision computation pipeline with the global seed (42).

Metric	Value
Label-agreement rate	0.971
Spearman ρ	0.819
Pearson r	0.906
Coefficient of determination (R^2^)	0.269

**Table 16:** Error profiles for the hybrid stacked ensemble on the internal test set. Each row corresponds to a misclassified sample; feature values are reported for the robust scale used by the model.

True Label	Predicted Prob.	Error Type	Mean Radius	Mean Concavity	Worst Concave Points
1	0.098	False Negative	0.272	0.582	0.234
1	0.100	False Negative	−0.252	0.464	0.477
1	0.404	False Negative	0.490	0.501	0.494
0	0.621	False Positive	0.466	−0.026	0.037
1	0.337	False Negative	0.059	0.244	0.191
1	0.174	False Negative	0.105	0.162	0.398
1	0.103	False Negative	0.301	0.221	0.373
1	0.391	False Negative	0.257	0.320	0.586

## Data Availability

The data presented in this study are openly available in Wisconsin Diagnostic Breast Cancer and Wisconsin Breast Cancer at https://archive.ics.uci.edu/ml/index.php (accessed on 4 May 2026).

## Abbreviations

The following abbreviations are used in this manuscript:

AUC	Area Under the Receiver Operating Characteristic Curve
CI	Confidence Interval
CNOT	Controlled-NOT gate
DCA	Decision Curve Analysis
ECE	Expected Calibration Error
F1	F1 Score
FN	False Negative
FP	False Positive
IECV	Internal–External Cross-Validation
LightGBM	Light Gradient-Boosting Machine
MCC	Matthews Correlation Coefficient
ML	Machine Learning
NISQ	Noisy Intermediate-Scale Quantum
QML	Quantum Machine Learning
QSVM	Quantum Support Vector Machine
RBF	Radial Basis Function
RFECV	Recursive Feature Elimination with Cross-Validation
ROC	Receiver Operating Characteristic
SD	Standard Deviation
SHAP	Shapley Additive Explanations
SVM	Support Vector Machine
TPE	Tree-Structured Parzen Estimator
TRIPOD AI	Transparent Reporting of a Multivariable Prediction Model for Individual Prognosis or Diagnosis—Artificial Intelligence
VQC	Variational Quantum Classifier
WBC	Wisconsin Original Breast Cancer Dataset
WDBC	Wisconsin Diagnostic Breast Cancer Dataset
XGBoost	Extreme Gradient Boosting
